# Genomic medicine for the 21^st^ century

**DOI:** 10.1308/rcsann.2024.0030

**Published:** 2024-04-01

**Authors:** I Rahman, J Barwell

**Affiliations:** University of Leicester, UK

There is currently a great deal of discussion regarding genomic medicine and how it will fundamentally change 21^st^ century healthcare if it is embraced and linked to a digital revolution. In this editorial and issue of the *Annals*, we ask what is genomics, how does it differ from clinical genetics and how can it be applied to surgical practice in a world beyond rare diseases?

**What is genomic medicine?**

**Figure 1:**
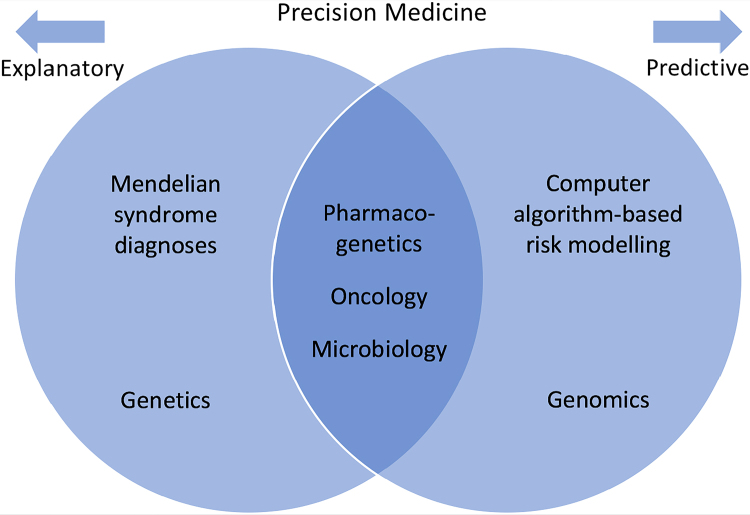
Interplay between different disciplines that constitute genomics

Genomic medicine is sometimes used interchangeably with whole genome sequencing, precision medicine or personalised medicine.^1^ Clinical genetics and genomics can be considered to overlap with personalised medicine as the cross-over area involving microbiology, pharmacogenetics, oncogenomics and targeted screening (Figure 1). Clinical genetics helps explain the reasons behind disease in families, personalised medicine predicts possible responses to planned management in individuals and genomics helps to define future risks through integrating complete datasets with health outcomes. This takes us from understanding our biological history through to mathematical algorithms that link our DNA code to our postcode but that can also integrate whole genome sequencing, radiology, biochemistry, body mass index, alcohol intake, phenotyping terms, and even sleep and exercise apps.

**The vision**

The goal is to identify patients with significant disease who require surgery at an earlier stage and to be better able to define the timing of preventative surgery based on calculated risk rather than age alone. Genomics aims to treat people based on the results of molecular tests and computer algorithms supported by integrated datasets linked to health outcomes.

Integrated datasets are already use in CanRisk modelling for familial breast and ovarian cancer algorithms,^2^ and also for ten-year cardiovascular risk calculations in primary care.^3^

Single nucleotide polymorphism (SNP) analysis is an increasingly utilised tool in cardiovascular and breast cancer risk calculation (Table 1).^4,5^ SNPs are commonly variable sequence changes at a single base and occur at about one in a thousand base intervals. These common landmarks can be used to identify us^6^ and to carry out a higher resolution (more detailed) genomic analysis^7^ than the light microscope is able to achieve. Many of these SNPs when combined may also provide information on the risk of disease either because they lie in the coding region of genes or because they alter their expression in some way that alters molecular mechanisms and influences risk.^8,9^

**Table 1:** Types of genomic tests and requirements

**Tests** Single nucleotide polymorphisms Genomic signatures Whole genome sequencing of blood and tumours Cell-free DNA Integrated computer modelling Microbiome analysis Pharmacogenetics
**Requirements** Genomic and phenotyping databases Standardised test directory Standardised variant interpretation mechanism Standardised consent forms and training National Genomic Research Library Patient participation forms

**The 100,000 Genomes Project catalyst**

The catalyst for introducing the infrastructure to deliver this was the Olympic Games legacy 100,000 Genomes Project^10,11^ and its central pillars of:

• standardisation of genetics testing criteria and variant analysis;
• consolidation of laboratories;
• democratisation of testing through mainstreaming and standardisation of consent forms;
• use of linked phenotyping databases so that comparisons can be made with other patients;
• the introduction of whole genome sequencing of both fresh tumour samples for planning treatment/understanding possible inherited susceptibility traits, and paired affected child and parent blood samples for germline comparative trio analysis to in order to find *de novo* pathogenic variants to reduce the length of the diagnostic odyssey for complex neurometabolic problems;
• improved collaboration with patients, stakeholders and research groups to ensure that data are used appropriately and effectively;
• Health Education England supported teaching and training.

**National infrastructure**

The 100,000 Genomes Project has led to the development of seven Genomic Laboratory Hubs^12^ offering the genomics test directory with the support of clinical genetics, mainstream specialties, training teams and patient groups via seven regional Genomic Medicine Service Alliances,^13^ which are tasked with ensuring that these are delivered in an efficient and equitable manner to improve patient outcomes.

**Where might you first encounter genomics?**

Genomics has the potential to identify individuals who are at risk and eligible for surgery through genetic testing and integrated computer modelling but it is also starting to be used in novel population screening approaches (Table 2). One example is the Galleri liquid biopsy blood test set up at supermarkets to help detect a higher proportion of tumours at stage 1 and 2, thereby improving cancer survivorship.^14^ The Galleri test assesses methylation signatures that are released in cell-free DNA from tumours into the plasma for 50 of the most common tumours, with a positive predictive value of 44% and a false positive rate of 0.5%.^15^ Early data suggest this will pick up significant and asymptomatic disease but not necessarily detect a high proportion of tumours at stage 1 and 2. This is vital as detecting tumours earlier allows quicker surgical intervention for several types of cancers including breast and colorectal cancer.

**Table 2:** Genomic presentations

Galleri liquid biopsies for cancer investigations
Computerised risk modelling for preventative surgery
Consenting for tumour or germline testing for treatment
Use of prognostic markers for decisions around chemotherapy or cell-free DNA to track tumour evolution
Titrating prescription doses and use of molecular care pathways

**Mainstreaming of genomic testing**

Mainstreaming involves consenting for blood tests to detect inherited disease susceptibility (such as breast or bowel cancer) in secondary care with training and downstream support from clinical genetics services. With an expansion in the number of eligible patients for testing and the rise of new technologies, it will be difficult for a limited cancer genetics consultant workforce to lead consent, testing, result interpretation and ongoing management for all patients across every multidisciplinary team (MDT). Dedicated testing hubs and specialised nurses supported by education and information governance systems will be required to support surgeons with complex results discussed at regional genomic MDT meetings.

Mainstreaming of genomic testing includes nationally agreed and standardised focused germline (blood) and somatic tissue gene panel testing or genomic signature assays.^16^ These signatures might include whole genome sequencing, often on frozen tumour samples, or microsatellite instability assays for Lynch syndrome,^17^ or homologous recombination deficiency assays or *BRCA*-like genomic instability^18^ on paraffin fixed blocks. There are approved and nationally agreed consent forms for DNA extraction, storage and use; these state the potential impact on family members and the possibility of finding unclear variants or incidental findings beyond the original remit of the test. In relation to surgery, this is crucial in mainstreaming genetic tests such as *BRCA1*, *BRCA2* and *PALB2* testing in a familial breast cancer setting or Lynch syndrome testing for patients with colorectal adenocarcinoma.

**Tracking the biological history of tumours**

Apart from the Galleri test being used in cancer detection, there has been an increasing use of serial non-invasive cell-free DNA testing in cancer patients.^19^ Although it is currently unable to distinguish between or detect all cancers, it will improve tracking tumour load (the number of cancer cells, the size of a tumour or the amount of cancer in the body) and evolution, leading to possible change in treatment and screening requirements.

**Pharmacogenetics**

Dihydropyrimidine dehydrogenase (*DPYD*) genetic testing is already mandated before fluorouracil treatment^20^ and whole genome sequencing or pre-planned focused genetics testing provides an opportunity to automatically extract pharmacogenetic data so that we can reduce side effects and avoid ineffective prescribing for all commonly used medications in a similar way to avoiding allergies either through electronic prescribing alerts or patient help digital apps. As 99% of us have a significant and actionable variant in a commonly prescribed medication,^21^ this will be a major step towards mainstreaming genomics into everyday practice.

**Patient engagement**

Patient engagement and tackling disparities is vital in building trust in the collection and use of large datasets to define risk and ration future healthcare. The patient participation panel of the 100,000 Genomes Project^22^ has been pivotal in this, linked to media outreach campaigns and localised projects targeting particular communities that for various reasons may struggle to access new technologies for reasons relating to age, sex, sexual orientation, ethnicity or socioeconomic background.

Ethically, do we want our alcohol intake measurement to come from what we say to our doctor or our supermarket loyalty card? How A-level grade boundaries were calculated in 2020, relying partially on postcode, did not sit well with our sense of social justice^23,24^ and it is clear that technology will be curtailed not by what could be done but by what should be done. These issues need to be debated.

**Embracing research**

It is hoped that systematic construction of phenotyping and genotyping databases, consent forms, and projects such as Our Future Health^25^ and UK Biobank^25^ can build the necessary infrastructure to capitalise on the research potential created by these datasets. Companies such as Flatiron^26^ and TriNetX^27^ are offering hospital trusts opportunities to integrate datasets to identify specific patient cohorts, and also the potential to analyse international large datasets with artificial intelligence. In this way, every patient we encounter could improve our understanding of disease.

**The challenges**

Clinician time, training, IT and laboratory testing infrastructure, the unclear significance of detecting subtle genomic variants and the possibility of over-medicalisation are all valid concerns that will need to be addressed (Table 3).^28–30^ However, it is clear that treating all patients in the same way regardless of tumour or an individual's characteristics is completely medically inappropriate, harmful and wasteful of precious and limited resources, and there will be a smarter way of delivering 21^st^ century healthcare.

**Table 3:** Factors relating to surgery and genomics that will drive progress

**Assets**	**Drivers**	**Blockers**	**Imperatives**
• Recent advances (Galleri study) • Our Future Health and UK Biobank • Commercial involvement • Genomics England databases • Health Education England resources	• Increasing patient autonomy and digital healthcare use after the COVID-19 pandemic • Personalised medicine • Concerns regarding medical harm due to failure to introduce pharmacogenetic testing • Cost of chemotherapy	• Access to training • Digital support • Laboratory capacity • Inequity of access • Clinician time • Understanding of the significance of some of the results • Tracking samples • Responsibility for decision making algorithms • Risk of over-medicalisation	• Standardised consent and time to discuss with patient • Coordination of research library • Tracking of samples and clarity on result placement • Governance to ensure results are managed appropriately • Access to education and training

**The response**

Research from the COVID-19 pandemic has seen patients use and submit data on apps, carry out tests at home, and discuss genomic variants, computer algorithms and clinical research findings over online forums and in the mainstream media. Clinical care is currently at the beginning of this molecular and digital journey, and clinical champions are needed from all specialties that are prepared to shape vision and make it a reality to improve patient outcomes.

Additional free online training for consent, inheritance patterns, common inherited syndromes, variant analysis and the principles behind new genome data visual representation systems such as genome wheels is available at:

• www.genomicseducation.hee.nhs.uk/genotes

• www.eastgenomics.nhs.uk/for-healthcare-professionals/genomic-tests/consent

• www.youtube.com/@ClinicalGenetics

• www.youtube.com/watch?v=aY7-VFqwzlY
